# Temperature Dependence of the Dielectric Function of Monolayer MoSe_2_

**DOI:** 10.1038/s41598-018-21508-5

**Published:** 2018-02-16

**Authors:** Han Gyeol Park, Tae Jung Kim, Farman Ullah, Van Long Le, Hoang Tung Nguyen, Yong Soo Kim, Young Dong Kim

**Affiliations:** 1 Department of Physics, Kyung Hee University, Seoul, 02447 Republic of Korea; 2 Center for Converging Humanities, Kyung Hee University, Seoul, 02447 Republic of Korea; 3 Department of Physics and Energy Harvest Storage Research Center (EHSRC), University of Ulsan, Ulsan, 44610 Republic of Korea

## Abstract

The dielectric function ${\boldsymbol{\varepsilon }}{\boldsymbol{=}}{{\boldsymbol{\varepsilon }}}_{{\bf{1}}}{\boldsymbol{+}}{\bf{i}}{{\boldsymbol{\varepsilon }}}_{{\bf{2}}}$ of monolayer molybdenum diselenide (MoSe_2_) is obtained and analyzed at temperatures from 31 to 300 K and at energies from 0.74 to 6.42 eV. The sample is a large-area, partially discontinuous monolayer (submonolayer) film of MoSe_2_ grown on a sapphire substrate by selenization of pulsed laser deposited MoO_3_ film. Morphological and optical characterizations verified the excellent quality of the film. The MoSe_2_ data were analyzed using the effective medium approximation, which treats the film and bare substrate regions as a single layer. Second derivatives of ε with respect to energy were numerically calculated and analyzed with standard lineshapes to extract accurate critical-point (CP) energies. We find only 6 CPs for monolayer MoSe_2_ at room temperature. At cryogenic temperatures 6 additional structures are resolved. The separations in the *B*- and *C*-excitonic peaks are also observed. All structures blue-shift and sharpen with decreasing temperature as a result of the reducing lattice constant and electron-phonon interactions. The temperature dependences of the CP energies were determined by fitting the data to the phenomenological expression that contains the Bose-Einstein statistical factor and the temperature coefficient.

## Introduction

Over the last few years, knowledge of two-dimensional (2-D) materials has rapidly expanded due to the enormous interest in nanotechnology and various unique physical and chemical properties based on dimensionality^1^. Group-VI transition-metal dichalcogenides (TMDs) are 2-D and MX_2_ materials, where M is a group-VI transition metal element (Mo or W) and X a chalcogen (S, Se, or Te). Common structural phases of TMDs include trigonal prismatic (2 *H*) and octahedral (1 *T*) coordinations of the metal atoms. Both phases form X-M-X layered structures coupled by weak van der Waals forces. This enables the fabrication of films of atomic thicknesses down to monolayers^2^.

TMDs are semiconducting materials, as opposed to graphene, which is metallic. Their bandgaps are tunable depending on the number of layers, tensile strain, and external electric field^3–5^. In addition most TMDs show a crossover from an indirect bandgap at multilayers to a direct bandgap at monolayers^3,6–8^. In particular, molybdenum diselenide (MoSe_2_) is a promising substitute for traditional semiconductors at the leading edge of research and technology for waveguiding, superconducting, and photodetecting devices^9–11^. The spin splitting of monolayer MoSe_2_ at the valence band maximum is larger than that of monolayer MoS_2_, making MoSe_2_ potentially useful for spintronic devices^3^.

The applications mentioned above are closely related to the dielectric properties of these materials. Spectroscopic ellipsometry (SE) is a precise and highly sensitive method for obtaining dielectric function data $\varepsilon ={\varepsilon }_{1}+i{\varepsilon }_{2}$^12^. Several workers have reported dielectric functions of monolayer MoSe_2_ using SE at room or high temperature, but these spectra are broad and transition peaks difficult to resolve^13,14^. To reduce thermal broadening, enhance weak features, and improve resolution, data must be obtained at cryogenic temperatures^15–17^. Furthermore, temperature dependences are needed to fully understand the working principles of devices and to properly design them for applications^18^. Although absorption data from 77 to 300 K in the energy region of the *A*- and *B*-excitonic peaks have been published, a systematic analysis of $\varepsilon$ of monolayer MoSe_2_ from cryogenic to room temperature has not been reported^18,19^.

Here, we report $\varepsilon$ data for monolayer MoSe_2_ from 0.74 to 6.42 eV at temperatures from 31 to 300 K. The sample was a large area (15 × 15 mm^2^) submonolayer MoSe_2_ film grown by selenization of MoO_3_ film that was deposited on the polished side of a sapphire substrate using pulsed laser deposition (PLD). Data were obtained by SE and analyzed using the Bruggeman effective medium approximation (EMA)^20^. Standard lineshape analysis was done using numerically calculated second derivatives of $\varepsilon$ with respect to energy to extract accurate critical-point (CP) energies. Six CPs are observed at room temperature. Six additional CPs are found at cryogenic temperatures. The *A*-excitonic peak at 1.61 eV shows a distinct shoulder at low temperature, which is due to the negatively charged *A*-excitonic (*A*-trionic) peak^19,21^. At 31 and 50 K, a CP is found between the *A*- and *B*-excitonic peaks. We interpret this as either a *B*-trionic peak^22^ or the first excited state of the *A*-exciton^23^, as predicted theoretically. The separation of the *C*-excitonic peaks, which has not reported so far, results from the spin-orbit splitting of the top valence band^24^. The CPs blue-shift and the associated features become more prominent with decreasing temperature as a result of the reduction of lattice constant and electron-phonon interactions^15^. The temperature dependences of the CP energies were determined by fitting the data to the phenomenological expression that contains the Bose-Einstein statistical factor and the temperature coefficient.

## Results and Discussion

### Synthesis and Characterizations

The procedure for preparing monolayer MoSe_2_ films is described in detail elsewhere^25^. Briefly, a krypton fluoride (KrF) excimer laser was used to deposit a thin film of MoO_3_ on the polished side of a sapphire substrate. The monolayer MoSe_2_ film was obtained by selenization of MoO_3_ in a selenium-rich environment in a two-zone hot-wall furnace. The chamber pressure and temperature were set to 450 mtorr and 900 °C, respectively. A mixture of Ar and H_2_ was employed as the carrier gas. A 25-min exposure ensured the conversion of MoO_3_ to MoSe_2_.

**Figure 1:**
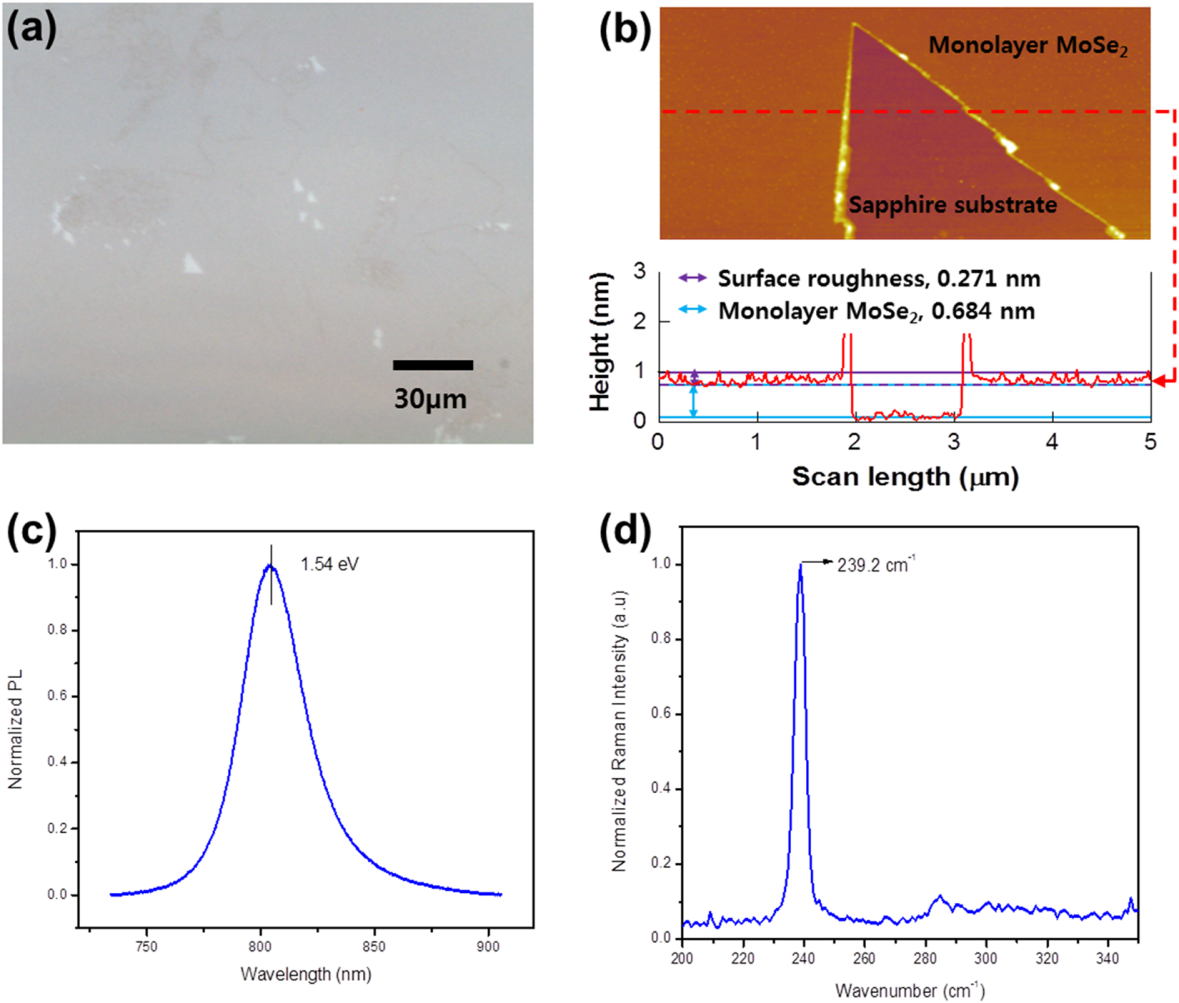
(**a**) OM image, (**b**) AFM image with thickness profile on red dashed line (inset), (**c**) PL spectrum, and (**d**) Raman spectrum of monolayer MoSe_2_ film deposited on the polished side of a sapphire substrate.

The MoSe_2_ film was characterized by optical microscopy (OM), atomic force microscopy (AFM), photoluminescence (PL), Raman spectroscopies, and X-ray diffraction. The OM image of the film is shown in Fig. 1a. The diameters of monolayer domains are a few hundred μm. The AFM image reveals that the grown MoSe_2_ film is of excellent quality with no adsorbed particles on its surface. The thickness is found to be 0.684 nm, confirming that the film is indeed a monolayer (Fig. 1b). Another hallmark of good quality is provided by the PL data. The PL intensity of semiconducting TMDs decreases exponentially with increasing thickness owing to the transition of the band gap from direct to indirect^8,11,26^. Our film shows a strong PL peak at ~800 nm (~1.55 eV), corresponding to the *A*-exciton, which arises from the *K*-point of the Billion zone (Fig. 1c)^21^.

The Raman spectra of monolayer MoSe_2_ is given in Fig. 1d. Two main peaks associated with the *A*_1g_ (out of plane) and *E*^1^_2g_ (in-plane) vibrational modes are observed at 239.2 cm^−1^ and 285.4 cm^−1^, respectively. The distance between the peaks, 46.2 cm^−1^, provided additional confirmation that film is monolayer MoSe_2_^27^. Moreover, the full-width at half-maximum (FWHM) of *A*_1g_ is 4 cm^−1^, indicating the highly crystalline nature of the layer^28^.

Several research groups have determined lattice parameters of TMDs using X-ray diffraction or first principles calculations. The ratio of the lattice parameters of MoSe_2_ and MoS_2_ is about 1.05^29,30^. In accordance with the ratio of lattice parameters and our previous work on MoS_2_, we concluded that the monolayer MoSe_2_ film is 0.693 nm thick. This agrees with thickness profile in Fig. 1b.

### SE measurement and analysis at room temperature

Because SE uses relatively weak continuum light sources, mm-scale beam illumination areas are required to obtain analyzable data, which is inconsistent with the sizes of the MoSe_2_ grains. Consequently, there are only a few SE studies of monolayer MoSe_2_. We circumvented the size/intensity challenge using a focusing probe, as described in Methods. This allowed us to obtain spectra of domains several hundred μm in size at room temperature.

The measured pseudodielectric functions $< \varepsilon  > = < {\varepsilon }_{1} > +i < {\varepsilon }_{2} >$ encode all sample information over the entire penetration depth of light, including not only the dielectric function of the film but also its thickness and the dielectric function of the substrate. To extract the dielectric function of the film, we used a four-phase optical model consisting of the ambient, a rough surface, the MoSe_2_ monolayer, and the sapphire substrate. This is denoted here as the RT model.

The $\varepsilon$ of monolayer MoSe_2_ was modelled using Gaussian and Tauc-Lorentz (TL) oscillators. ${\varepsilon }_{2}$ of the Gaussian oscillator^31^ is symmetric:

1 $${\varepsilon }_{2{\rm{G}}}={A}_{{\rm{G}}}{e}^{-{(\frac{E-{E}_{{\rm{G}}}}{\sigma })}^{2}},\,{\rm{\sigma }}=\frac{Br}{2\sqrt{\mathrm{ln}(2)}}\,,$$

where *A*_G_ is the amplitude, *E*_G_ is the central energy, and *Br* is FWHM. ${\varepsilon }_{2}$ of the TL oscillator^32^ is asymmetric:

2 $$\begin{array}{cc}{\varepsilon }_{2{\rm{T}}{\rm{L}}} & =\,[\frac{{A}_{{\rm{T}}{\rm{L}}}{E}_{{\rm{T}}{\rm{L}}}C{(E-{E}_{{\rm{g}}{\rm{a}}{\rm{p}}})}^{2}}{{({E}^{2}-{E}_{{\rm{T}}{\rm{L}}}^{2})}^{2}+{C}^{2}{E}^{2}}\cdot \frac{1}{E}],\,E > {E}_{{\rm{g}}{\rm{a}}{\rm{p}}},\\  & =\,0\quad \quad \quad \quad \quad \quad \quad \quad \,,\,E\le {E}_{{\rm{g}}{\rm{a}}{\rm{p}}},\end{array}$$

where *A*_TL_ is the amplitude, *E*_TL_ is the central energy, *C* is the broadening parameter, and *E*_gap_ is the Tauc gap. ${\varepsilon }_{1}$ was calculated from ${\varepsilon }_{2}$ with the Kramers-Kronig integral. In general, a combination of Gaussian and TL oscillators reproduces the dielectric lineshape of monolayer MoSe_2_ fairly well, but a point-by-point analysis is needed to obtain CP energies, as done below.

The dielectric spectrum of the rough-surface layer is represented in the Bruggeman EMA^20^ modeled as a mixture of 50% monolayer MoSe_2_ and 50% ambient. Our best fit to the monolayer-MoSe_2_ data at room temperature yielded a 0.284 nm thickness for the rough interface. This is consistent with the AFM result in Fig. 1b.

**Figure 2:**
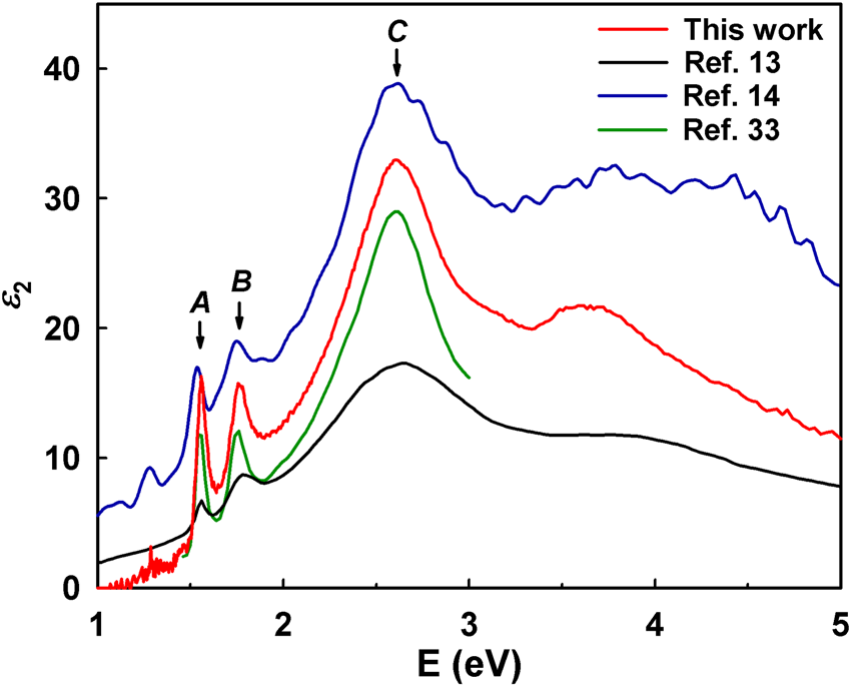
Imaginary parts of dielectric spectra of monolayer MoSe_2_ at room temperature compared to data previously reported in refs^13,14,33^.

Figure 2 compares our ${\varepsilon }_{2}$ results for a monolayer MoSe_2_ domain at room temperature with previously reported data^13,14,33^. Structureless regions at energies less than 1 eV and more than 5 eV are deleted for clarity. All data reproduce the *A*, *B*, and *C* features, but considerable variation is seen above 3 eV. For monolayer-scale films, such differences arise from discrepancies between the actual thickness of a film and the thickness assumed upon inverting the thin-film model. Thus thicknesses must be carefully assessed by different techniques to ensure consistency and hence accuracy, as done here. The noisy feature at 1.24 eV seems to be an artifact caused by change of detectors, as explained in Methods below.

### SE measurement and analysis at various temperatures

To acquire temperature dependences, the MoSe_2_ sample was placed on a cold-finger sample holder with silver paste and sealed in an ultrahigh vacuum cryostat chamber^17^, as described in Methods. Condensation artifacts at low temperatures were therefore minimized. Stress-free fused-quartz windows on the chamber provided non-normal-incidence optical access to the sample^34^. The temperature of the MoSe_2_ sample was decreased to 31 K with the closed-cycle helium refrigerator. $< \varepsilon  >$ was determined from 0.74 to 6.42 eV at 31 K and at 25 K intervals starting at 300 K. The data themselves were acquired with a dual-rotating-compensator SE without the focusing probe, as described in Methods. Therefore, the measured $< \varepsilon  >$ for the temperature dependence study contain not only the information about the perfect monolayer domains but also of the incomplete-monolayer and substrate-exposed areas.

$< \varepsilon  >$ was analyzed using a 5-phase optical model consisting of the ambient, a rough surface, the submonolayer MoSe_2_ film, the sapphire substrate, and the silver paste backing. We call it TD model. We analyzed the data assuming coherent superposition for the front-surface reflection and incoherent superposition for the back-surface reflection. The back-surface contribution therefore affects the intensity not the polarization state. The back side of our substrate was unpolished, and so its contribution to the intensity is small, and the measured depolarization is less than 1.5% over the entire spectral range. The $\varepsilon$ of submonolayer is modeled by Bruggeman EMA comprised of monolayer MoSe_2_, the defect region, and the ambient. The $\varepsilon$ of monolayer MoSe_2_ is the red line in Fig. 2. The broad spectrum of the defect region was determined by averaging several measurements on incomplete MoSe_2_ and MoO_3_ areas using a focused beam spot (Fig. S1). The material ratio in the EMA was found to be 54.53% monolayer MoSe2, 27.34% defect region, and 18.13% ambient. A point-by-point approach was used to extract $\varepsilon$ of the monolayer MoSe_2_ from the TD model. The two $\varepsilon$ spectra deducted from the RT and TD models at room temperature agree well, as shown in Fig. S2. We carefully examine these dielectric spectra in next section. The point-by-point approach on TD model was applied to the other data at various temperatures with same parameters, except for the thicknesses of the MoSe_2_ and roughness layers, since the film thickness decreases with temperature. We took this into consideration from literature X-ray data and first principles calculations of MoSe_2_ lattice parameters^35,36^. The ratio of thicknesses obtained by X-ray scattering at temperatures 77 K and 300 K is 1.0015, a difference of less than 0.2%, having estimated thickness of MoSe_2_ layerat 31 K is 0.692 nm.

**Figure 3:**
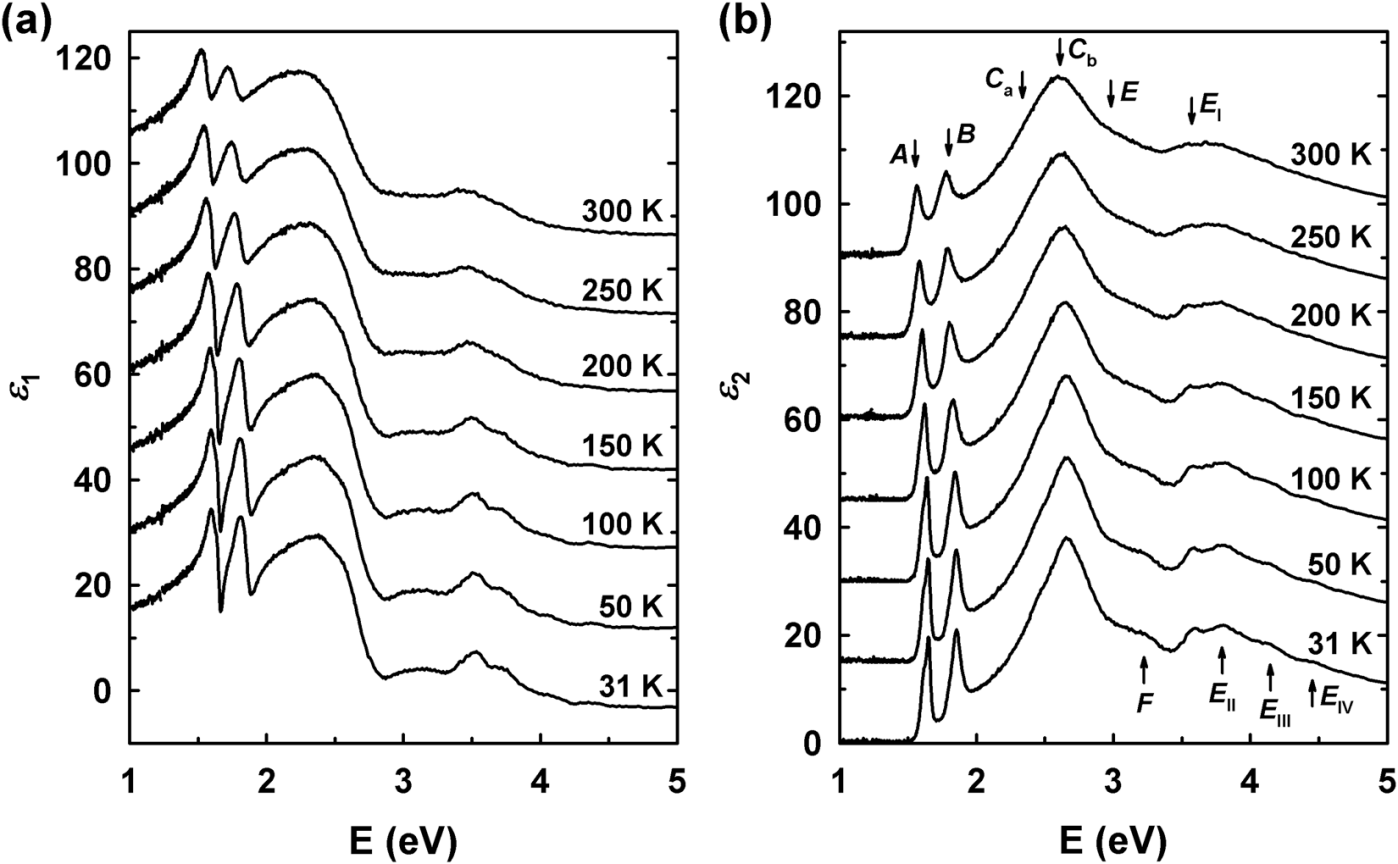
(**a**) Real and (**b**) imaginary parts of $\varepsilon$ of monolayer MoSe_2_ at temperatures from 31 to 300 K. Data from 50 K are in steps of 50 K. The spectra are offset by increments of 15 relative to the 31 K spectrum.

Figure 3 shows our $\varepsilon$ results for monolayer MoSe_2_ at temperatures from 31 to 300 K offset by increments of 15 relative to the 31 K spectrum. 10 CP structures (*A*, *B*, *C*_a_, *C*_b_, *E*, *F*, and *E*_I-IV_) are marked in Fig. 3(b). The identification of *A*, *B*, *C*_a_, *C*_b_, *E*, and *F* CPs is determined by band-structure calculations. The *A* and *B* CPs, and the *C*_a_ and *C*_b_ CPs, involve transitions from the top of the valence band and its spin-orbit-split partner to the lowest conduction band at *K* and the *X* (around $\frac{2}{3}$ of Γ-*K* line; $\vec{k}=\frac{2}{3}(\frac{4\pi }{3a}{\hat{k}}_{x})\,({m}^{-1})$), respectively^23,24^.

The main contributions of the *E* CP arise from multiple transitions near the $$\frac{1}{2}$$ *K*-*M* line (; $$\vec{k}=\frac{1}{2}(\frac{7\pi }{3a}{\hat{k}}_{x}-\frac{\pi }{\sqrt{3}a}{\hat{k}}_{y})\,$$$$({m}^{-1})$$) and the *X* point^24^. We find five additional CPs (*F* and *E*_I-IV_) above 3 eV that have not yet been reported. The origin of the *F* CP structure might be understood as E2^+/−^ multiple transitions between Γ and *K* points according to ref.^24^, since the energy difference of about 0.3 eV between the main contributions of the *E* and *F* CPs matches the difference between the E1^+/−^ and E2^+/−^ transitions in ref.^24^, where the ‘ + ’ and ‘−’ symbols indicate that the transitions are split by spin-orbit interaction. Since the transition points of most CPs of monolayer MoSe_2_ are fairly similar to those of MoTe_2_^24^, the *E*_I_ and *E*_II_ CPs can be assigned to transitions around the $$\frac{1}{2}$$ *M*-Γ line or the *M*-point, where the G^+/−^ and H^+/−^ transitions occur in monolayer MoTe_2_. Since these conjectures do not consider density-of-states and transition-probability, a further systematic theoretical investigation is needed. All CP structures blue-shift and sharpen as the temperature was lowered. This is consistent with the reduced lattice parameter and electron-phonon interaction at low temperatures^15^.

**Figure 4:**
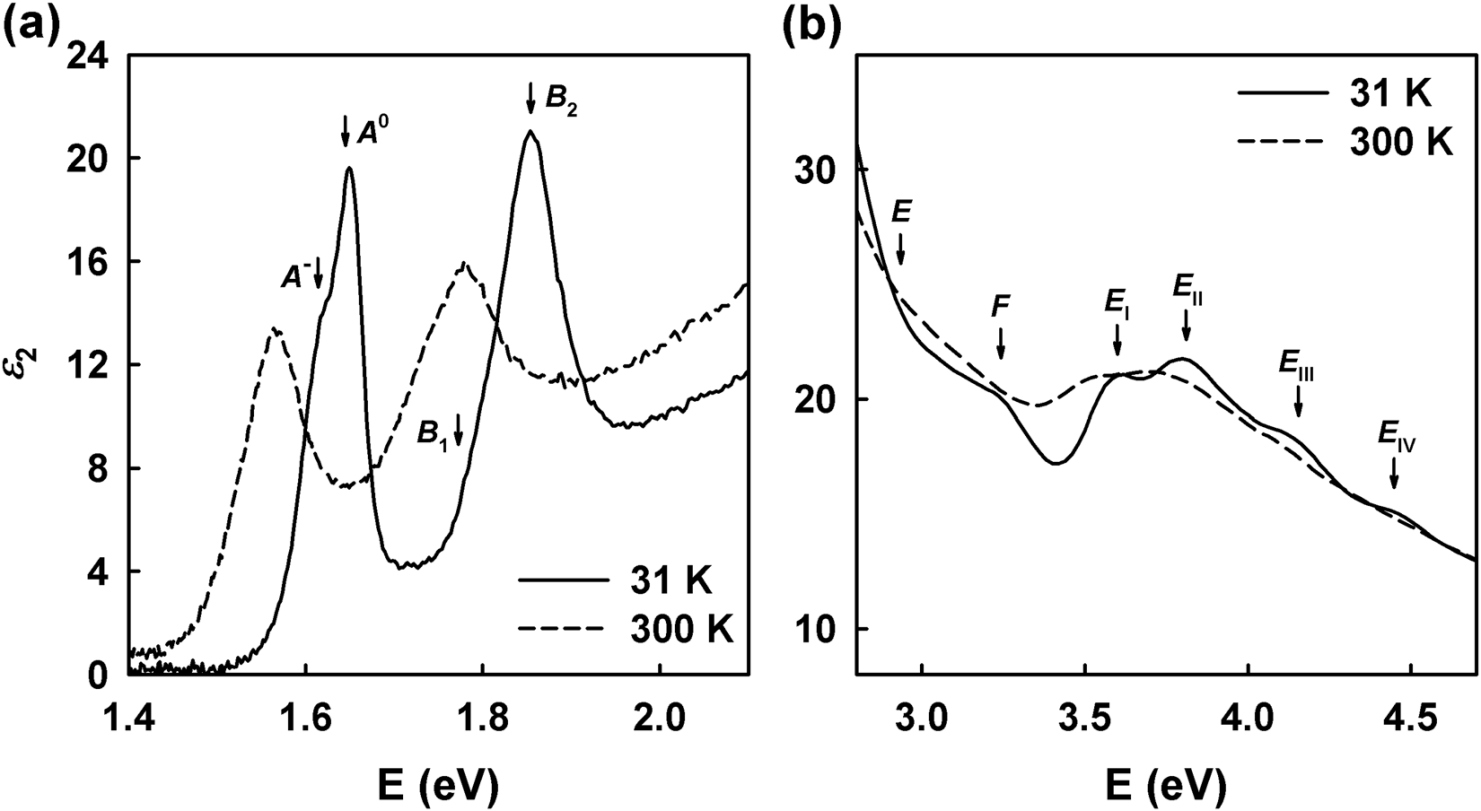
${\varepsilon }_{2}$ spectra of monolayer MoSe_2_ at 31 (solid line) and 300 K (dashed line) on expanded scales near regions of (**a**) *A*^−^, *A*^0^, *B*_1_, and *B*_2_ CPs and (**b**) *E*, *F*, and *E*_I-IV_ CPs.

For detailed analysis, ${\varepsilon }_{2}$ at 31 and 300 K is plotted on expanded scales in two ranges in Figs 4a and b. A linear filtering algorithm of Savitzky and Golay (Table I of ref.^38^, 11-point convolution) was used to highlight distinct CP structures in Fig. 4(b). The 31 K data exhibit a clear shoulder in the *A* exciton at about 1.6 eV. This is interpreted in temperature-dependent absorption and PL studies on monolayer MoSe_2_^19,21^ to originate from a tightly bound charged exciton. The *B* peak shows no obvious shoulder, but has a strongly asymmetric lineshape that cannot be described by a single oscillator. A band-structure calculation of MoSe_2_ anticipated another transition in the *B*-excitonic peak, interpreting it as the first excited state of the *A* exciton^23^. Excited exciton states are observed in monolayer WS_2_^37^, but have not yet been reported for monolayer MoSe_2_. However, another band-structure calculation on similar material of monolayer MoS_2_ indicates the existence of a *B*-trionic peak next to the *B*-exitonic peak^22^. The identification of the *B*_1_ CP is not yet clear because it is dominated by the huge *B*_2_ CP. This needs to be investigated in more detail in the future.

### CP Analysis

Second derivatives $\frac{{d}^{2}\varepsilon }{d{E}^{2}}$ were evaluated numerically to distinguish overlapping CP structures and to determine values of CP energies. These calculations were done with a Savitzky-Golay second-derivative algorithm (Table IV of ref.^38^, 11-point convolution) to reduce noise and minimize lineshape distortion^38^. Regression analysis was then used to fit CP parameters to the standard analytic CP expression^39^

3 $$\begin{array}{c}\frac{{d}^{2}\varepsilon }{d{\omega }^{2}}=n(n-1){A}_{{\rm{amp}}}{e}^{i\varphi }{(\hslash \omega -E+i{\rm{\Gamma }})}^{n-2},\,n\ne 0,\\ \quad \quad =\,{A}_{{\rm{amp}}}{e}^{i\varphi }{(\hslash \omega -E+i{\rm{\Gamma }})}^{-2}\,,\,n=0,\end{array}$$

where *A*_amp_ is the amplitude, *ϕ* is the phase, *E* is the CP energy, and Γ is the broadening parameter. The exponent *n* = $-1,\,-\frac{1}{2},\,0,\,\mathrm{and}\,\frac{1}{2}$ describes excitonic, and one-, two-, and three-dimensional CPs, respectively. In the 2-D system, *n* = $\frac{1}{2}$ is therefore forbidden. We simultaneously fit real and imaginary parts of $\frac{{d}^{2}\varepsilon }{d{E}^{2}}$ to better define the parameters. The excitonic lineshape ($n=-1$) yields the best representation of all CPs of the monolayer material. This was also the case for MoS_2_^17,40^.

**Figure 5:**
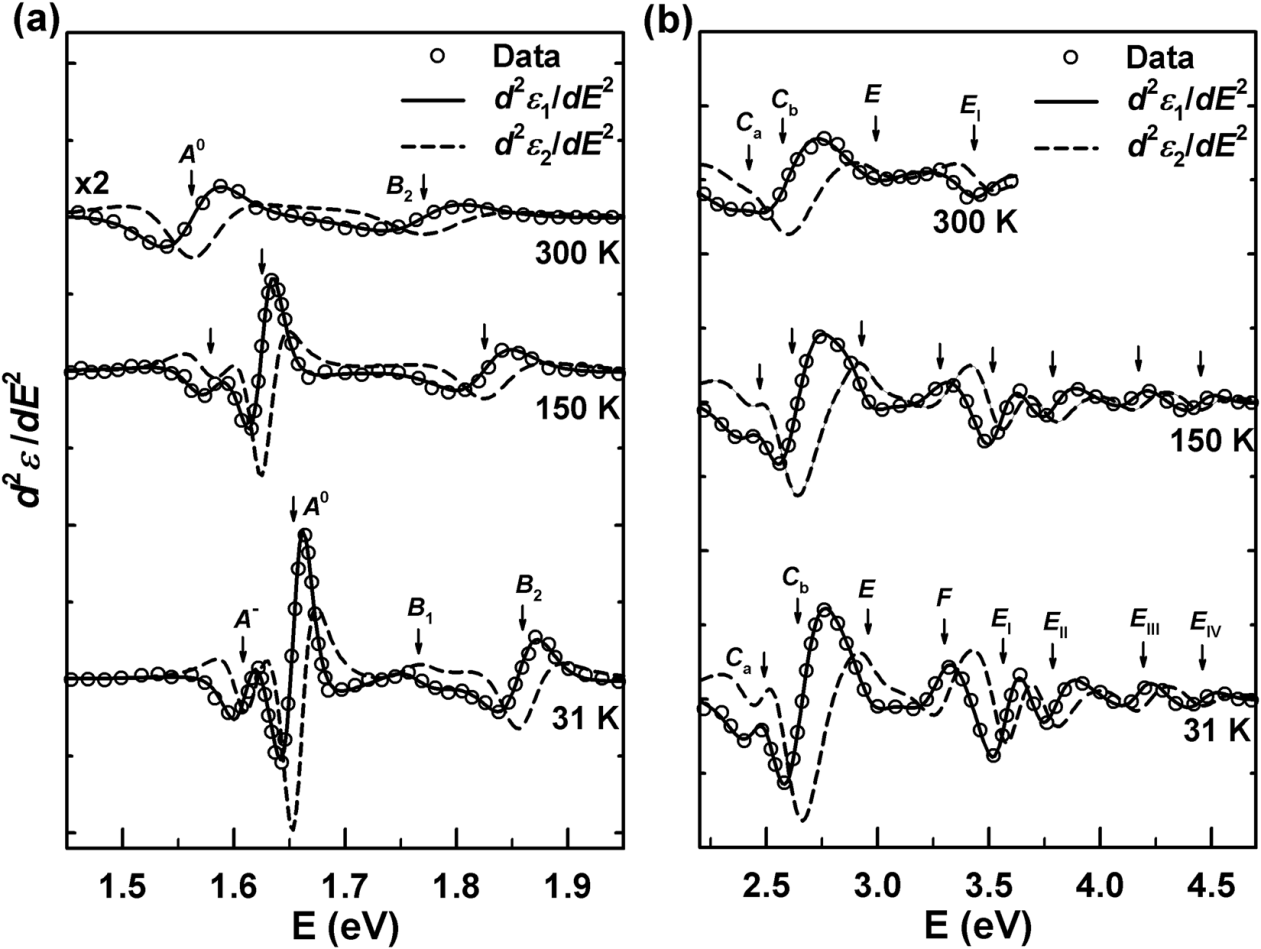
Second energy derivatives of $\varepsilon$ of monolayer MoSe_2_ at 31, 150, and 300 K for (a) *A*^−^, *A*^0^, *B*_1_, and *B*_2_ CPs and (b) *C*_a_, *C*_b_, *E*, *F*, and *E*_I-IV_ CPs. The best fits are to $\frac{{d}^{2}{\varepsilon }_{1}}{d{E}^{2}}$ (solid lines) and $\frac{{d}^{2}{\varepsilon }_{2}}{d{E}^{2}}$ (dashed lines). For clarity, only $\frac{{d}^{2}{\varepsilon }_{1}}{d{E}^{2}}$ (open circles) is shown, and the number of points is appropriately reduced.

Figure 5 shows the second derivatives and their best fits at 31, 150, and 300 K. The open circles are the calculated second derivatives of ${\varepsilon }_{1}$, and the solid and the dashed lines the best-fit results for $\frac{{d}^{2}{\varepsilon }_{1}}{d{E}^{2}}$ and $\frac{{d}^{2}{\varepsilon }_{2}}{d{E}^{2}}$, respectively. The data for $\frac{{d}^{2}{\varepsilon }_{2}}{d{E}^{2}}$ are not shown. The number of points for $\frac{{d}^{2}{\varepsilon }_{1}}{d{E}^{2}}$ was properly reduced for clarity. In the derivative spectra, CP energies blue-shift as the temperature is reduced, and the 31 K CP structures are obviously larger and sharper than those determined for higher temperatures. Near room temperature the *E*_II-IV_ CPs cannot be analyzed due to the low signal-to-noise ratio. Thus the high-energy region above 3.62 eV eliminated to prevent erroneous conclusions about overlapping CP.

The exact values of CP energies at 31 and 300 K from the best fits are listed on Table 1. Most match previously reported values^13,23,24,33^. The *B*_1_ and *E*_II-IV_ CP energies have not been reported so far.

**Table 1:** CP energies at 31 and 300 K compared to data previously reported in refs^13,23,24,33^.

CP energies (eV)	This work		Experiment (RT)		Band Calculation	
	31 K	300 K	SE^13^	Reflectance^33^	GW-BSE^23^	GW-BSE^24^
*A* ^−^	1.61	—	1.62	1.55^b^	1.61	1.86
*A* ^0^	1.66	1.56				
*B*_1_	1.76	—	1.82	1.75	1.82^c^	2.03
*B*_2_	1.86	1.77				
*C*_a_	2.49	2.43	—	—	2.25^c^	2.45
*C*_b_	2.64	2.57	2.64^a^	2.62^b^	2.35^c^	2.55
*E*	2.95	3.00	—	—	—	2.76^d^
*F*	3.30	—	—	—	—	3.00^d^
*E*_I_	3.56	3.44	—	—	—	—
*E*_II_	3.77	—	—	—	—	—
*E*_III_	4.19	—	—	—	—	—
*E*_IV_	4.46	—	—	—	—	—

^a^Extracted from Fig. 1(b) of ref.^13^.

^b^Extracted from Fig. 1(i) of ref.^33^.

^c^Extracted from Fig. 4(c) of ref.^23^.

^d^Extracted from Fig. 1(c) of ref.^24^.

As mentioned above, the $\varepsilon$ from TD model was carefully compared with the result of RT model using CP analysis (Fig. S3, Table S1). The differences of 6 CP (*A*^0^, *B*_2_, *C*_a_, *C*_b_, *E*, and *E*_I_) energies at 300 K between two models are under 0.03 eV which are generally less than the differences among the reported CP energies^13,23,24,33^.

**Figure 6:**
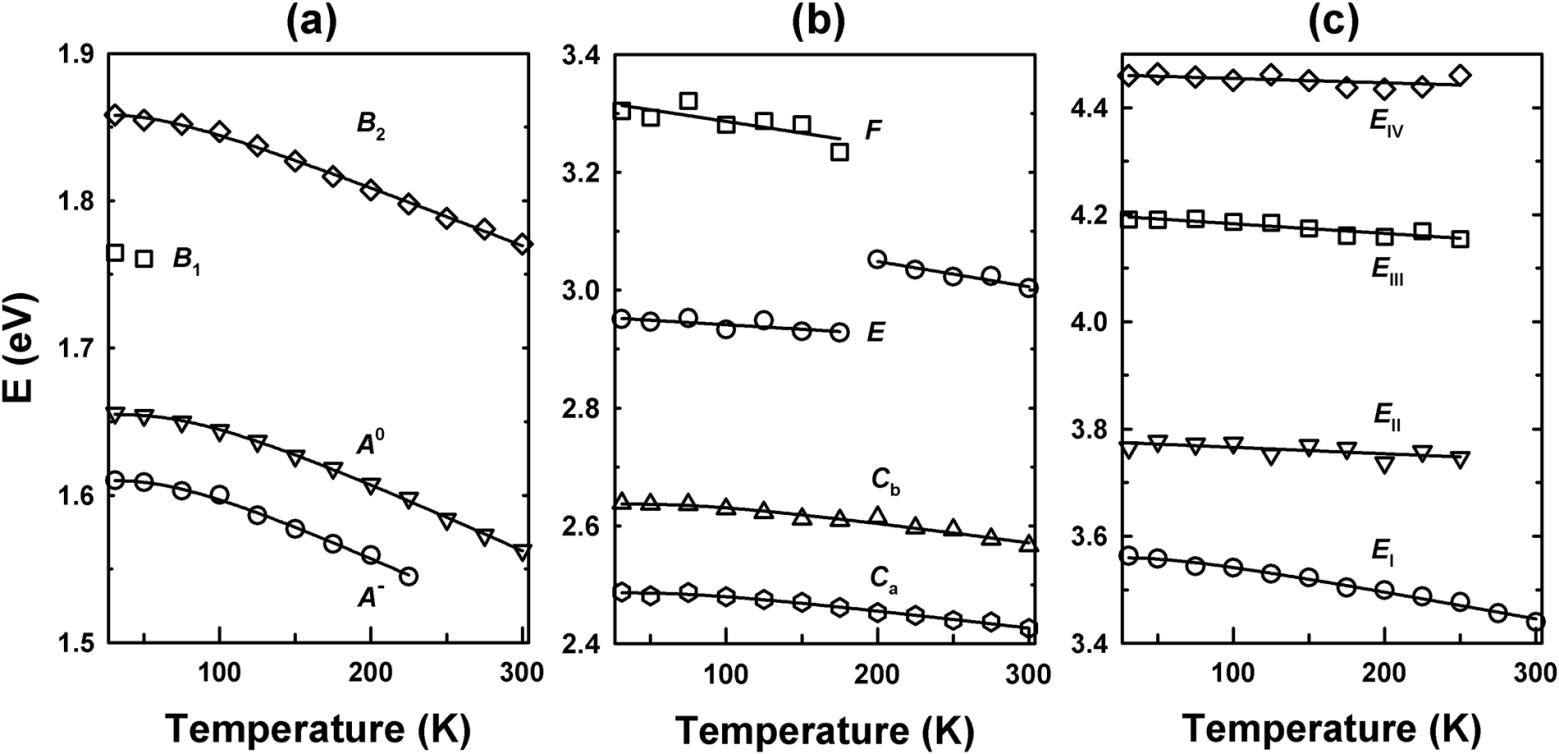
Temperature dependences of the CP energies (open symbols) of monolayer MoSe_2_ and the best fits (solid lines) for (**a**) *A*^−^, *A*^0^, *B*_1_, and *B*_2_ CPs, (**b**) *C*_a_, *C*_b_, *E*, and *F* CPs, and (**c**) *E*_I-IV_ CPs.

The temperature dependences of the CPs are shown in Fig. 6. The open dots are CP energies from the regression analysis and the solid lines are best fits using either a phenomenological expression or a linear equation. *A*^−^, *A*^0^, *B*_2_, *C*_a_, *C*_b_, and *E*_I_ CPs were fit with the expression that contains the Bose-Einstein statistical factor for phonons^16^:

4 $$E(T)={E}_{{\rm{B}}}-{a}_{{\rm{B}}}[1+\frac{2}{{e}^{{\rm{\Theta }}/T}-1}],$$

where *E*_B_, *a*_B_, and Θ, are CP energy at 0 K, strength of electron-phonon interaction, and mean frequency of phonons, respectively. *E*, *F*, and *E*_II-IV_ CPs were fit with the linear equation^16^:

5 $$E(T)={E}_{{\rm{L}}}-\lambda T,$$

where *E*_L_ is CP energy at 0 K and -*λ* is the temperature coefficient, *dE*/*dT*. In Fig. 6a, the *A*^−^ and *A*^0^ CPs have identical lineshapes, which certifies their adjoined transition origins. These two lineshapes are in good agreement with previously reported PL results^19,21^. (Fig. S4). Though there are mismatches caused by experimental uncertainties and differences between SE and PL, they are generally less than 0.02 eV. Therefore, the temperature dependences are reliable. The temperature dependence of *B*_1_ CP was not fit because the weak structure of *B*_1_ CP could only be resolved from that of the *B*_2_ CP at 31 and 50 K. As mentioned above, the *E* and *F* CPs appear to share transition points in the Brillouin zone. The relatively weak structure of the *F* CP was not separable from that of the *E* CP at temperatures above 180 K although the amplitude of the *F* CP is non-negligible. In Fig. 6b, the kink of the temperature dependence of *E* CP was caused by eliminating the overlapped *F* CP. Table 2 contains the best fit results with equation (4) or (5). The *a*_B_ and Θ values of *A*^−^ and *A*^0^ CPs are similar to each other, as expected from Fig. 6a. The Θ value of monolayer MoSe_2_ is probably less than that of bulk MoSe_2_, as expected for the Debye model in a lower dimension. Though the Θ value of bulk MoSe_2_ has not been reported, we can estimate it from values of Θ for monolayer and bulk MoS_2_ that are obtained from the phenomenological expression using ellipsometric and piezoreflectance data, respectively^17,41^. The Θ values of the *A*- and *B*-excitonic peaks of monolayer MoS_2_^17^ are about 250 K, while those of bulk MoS_2_ are about 550 K^41^. Therefore, we expect that the Θ values for monolayer MoSe_2_ given in Table 2 are physically consistent with reported values.

**Table 2:** Best-fit parameters for the temperature dependences of CP energies of monolayer MoSe_2_.

CPs	*E*_B_ (eV)	*a*_B_ (meV)	Θ (K)	*E*_*L*_ (eV)	λ (10^−4^ eVK^−1^)
*A* ^−^	1.66	54	221	—	—
*A* ^0^	1.72	63	258	—	—
*B*_2_	1.90	39	188	—	—
*C*_a_	2.53	42	260	—	—
*C*_b_	2.69	52	284	—	—
*E (31–175 K)*	—	—	—	2.96	1.54
*E* (200–300 K)	—	—	—	3.13	4.30
*F*	—	—	—	3.33	3.96
*E*_I_	3.61	48	183	—	—
*E*_II_	—	—	—	3.78	1.20
*E*_III_	—	—	—	4.20	1.82
*E*_IV_	—	—	—	4.46	0.81

## Conclusions

We report and analyze the intrinsic dielectric response *ε* of monolayer MoSe_2_ for energies from 0.74 to 6.42 eV and temperatures from 31 to 300 K. The data were obtained by SE. 6 CPs (*A*^0^, *B*_2_, *C*_a_, *C*_b_, *E*, and *E*_I_) at 300 K and 6 additional CPs (*A*^−^, *B*_1_, *F*, and *E*_II-IV_) at cryogenic temperatures were observed. In particular, the appearance of separate *B*_1_ and *B*_2_, and *C*_a_ and *C*_b_ CPs were expected from theoretical studies only. Numerically calculated second derivatives of $\varepsilon$ with respect to energy were used to extract CP energies. At low temperatures, sharper structures at higher energies were observed as the result of decreased lattice constant and electron-phonon interactions. We determined the temperature dependence of each CP energy using a linear equation or a phenomenological expression that contains the Bose-Einstein statistical factor. This allows CP energies to be estimated at every temperature from 31 to 300 K. These results are helpful for understanding the optical characteristics of monolayer MoSe_2_, and to facilitate precise engineering of optoelectronic devices based on 2-D materials.

## Methods

### Monolayer MoSe_2_ growth

The sample was prepared by PLD followed by selenization. A highly pure MoO_3_ target of approximately 20 mm diameter was placed in a rotating target holder. The target was exposed to a laser beam for several minutes to remove surface contaminants. A sapphire substrate approximately 20 × 20 mm^2^ was placed in a rotating substrate holder. The distance between the substrate and target was 25 cm. The chamber pressure was 25 mTorr, established by flowing high pure argon (Ar) at 10 sccm. A KrF excimer laser (λ = 248 nm, CompexPro 102 F) with 20 ns pulse width, 200 mJ/cm^2^ power density, 3 Hz repetition rate deposited thin MoO_3_ films in 45 s. The substrate temperature during deposition was 700 °C. The as-deposited MoO_3_ films were then converted to MoSe_2_ by selenization in a two-zone hot-wall furnace. Se powder (Sigma Aldrich, 99.99%) was placed in the low temperature zone at 450 °C, and the film located in the high temperature zone at 900 °C. A mixture of Ar and H_2_ were employed as carrier gases to transport Se vapor from the colder to the hotter zone. H_2_ also assist in reducing MoO_3_.

### Characterization

PL and Raman spectra were obtained at room temperature using a diode-pumped solid-state laser (Lasos GmbH, BLK 73100 TS). Excitation was done at 473 nm with 10 mW power. The spot size of the laser was about ~10 μm. A three-grating monochromator (Dongwoo Optron, Monora 500i) with a focal length of 50 cm was used to analyze the light scattered from the sample. The detector was a thermoelectrically cooled Si CCD detector with a pixel matrix of 1024 × 244 (Andor Ultravac, iDus 420). Wavelengths were calibrated with a standard Ne calibration lamp (Newport, 6032).

Topographic images of the MoSe_2_ films were obtained using the intermittent contact mode of an AFM (Asylum Research, MFD-3D) with Si tips (Olympus, AC240). The nominal normal spring constant was 2 N/m and the scan speed was 0.5 Hz.

### SE

Pseudodielectric spectra of monolayer MoSe_2_ domains were recorded in the ‘Isotropic + Depolarization’ mode of a M2000-FI ellipsometer (J. A. Woollam Co., Inc.) The ellipsometer was equipped with a focusing probe accessory, which reduces the diameter of beam spot from ~10 mm to ~100 μm. The measurement range is 245 to 1000 nm at 1.5 nm intervals and from 1000 to 1664 nm at 3.5 nm intervals. Light sources were xenon and quartz tungsten halogen (QTH) lamps. The detectors were two charge-coupled device (CCD) arrays. Noise occurs near 1000 nm (~1.24 eV), where the Si detector loses sensitivity and is replaced with an InGaAs unit. Data were obtained at angles of incidence of 55, 60, 65, and 70°. Each data point represents an average of 500 revolutions of the compensator.

For cryogenic measurements, a different ellipsometer (RC2, J. A. Woollam Co., Inc.) was employed. This ellipsometer used QTH and deuterium lamps and Si and InGaAs CCD array detectors. Data were obtained at 1 nm intervals from 193 to 1000 nm, and at 2.5 nm intervals from 1000 to 1690 nm. The AOI was 68.457° and measurements were completed in 150 seconds. The focusing probe accessory was not used in the temperature-dependent experiments due to the lack of space between the cryostat chamber and the SE.

### Cryostat system

The cryostat consisted of a stainless-steel chamber with rotary and turbomolecular pumps, and operated near the 10^−8^ Torr vacuum level. The temperature of the MoSe_2_ sample was regulated using a 331 Temperature Controller (Lake Shore Cryotronics, Inc.) with a heating element inside the sample holder and a silicon-diode thermometer on the corner of the sample. A closed-cycle helium refrigerator (M125 Helium Compressor, Oxford Instruments plc) was employed for decreasing the temperature.

### Analysis of ***ε***

Rough modelling of $\varepsilon$ of monolayer MoSe_2_ was carried out by the WVASE software internal to the ellipsometers (version 3.826, J. A. Woollam Co., Inc.). with Tauc-Lorentz and Gaussian oscillators. Point-by-point approach to extract precise $\varepsilon$ of monolayer MoSe_2_ and its temperature dependence was also performed with WVASE program.

### Data availability

The datasets generated during and/or analyzed during the current study are available from the corresponding author on reasonable request.

## Electronic supplementary material

Supplementary Information
