# Supplementary material for: Temperature Dependence of the Dielectric Function of Monolayer MoSe2
Source: Sci Rep. 2018 Feb 16;8:3173. doi: 10.1038/s41598-018-21508-5 (PMC5816670; doi:10.1038/s41598-018-21508-5)
Supplement: Supplementary file 1 — Supplementary Information [file 41598_2018_21508_MOESM1_ESM.pdf]

Supplementary Information

for

Temperature Dependence of the Dielectric Function of Monolayer $MoSe_2$

Han Gyeol Park,[1] Tae Jung Kim,[2] Farman Ullah,[3] Van Long Le,[1] Hoang Tung Nguyen,[1] Yong Soo Kim,[3] and Young Dong Kim[1]

[1]Department of Physics, Kyung Hee University, Seoul 02447, Republic of Korea

[2]Center for Converging Humanities, Kyung Hee University, Seoul 02447, Republic of Korea

[3]Department of Physics and Energy Harvest Storage Research Center (EHSRC), University of Ulsan, Ulsan 44610, Republic of Korea

Correspondence and requests for materials should be addressed to T.J.K. (email: tjkim@khu.ac.kr), Y.S.K. (yskim2@ulsan.ac.kr), or Y.D.K. (ydkim@khu.ac.kr)

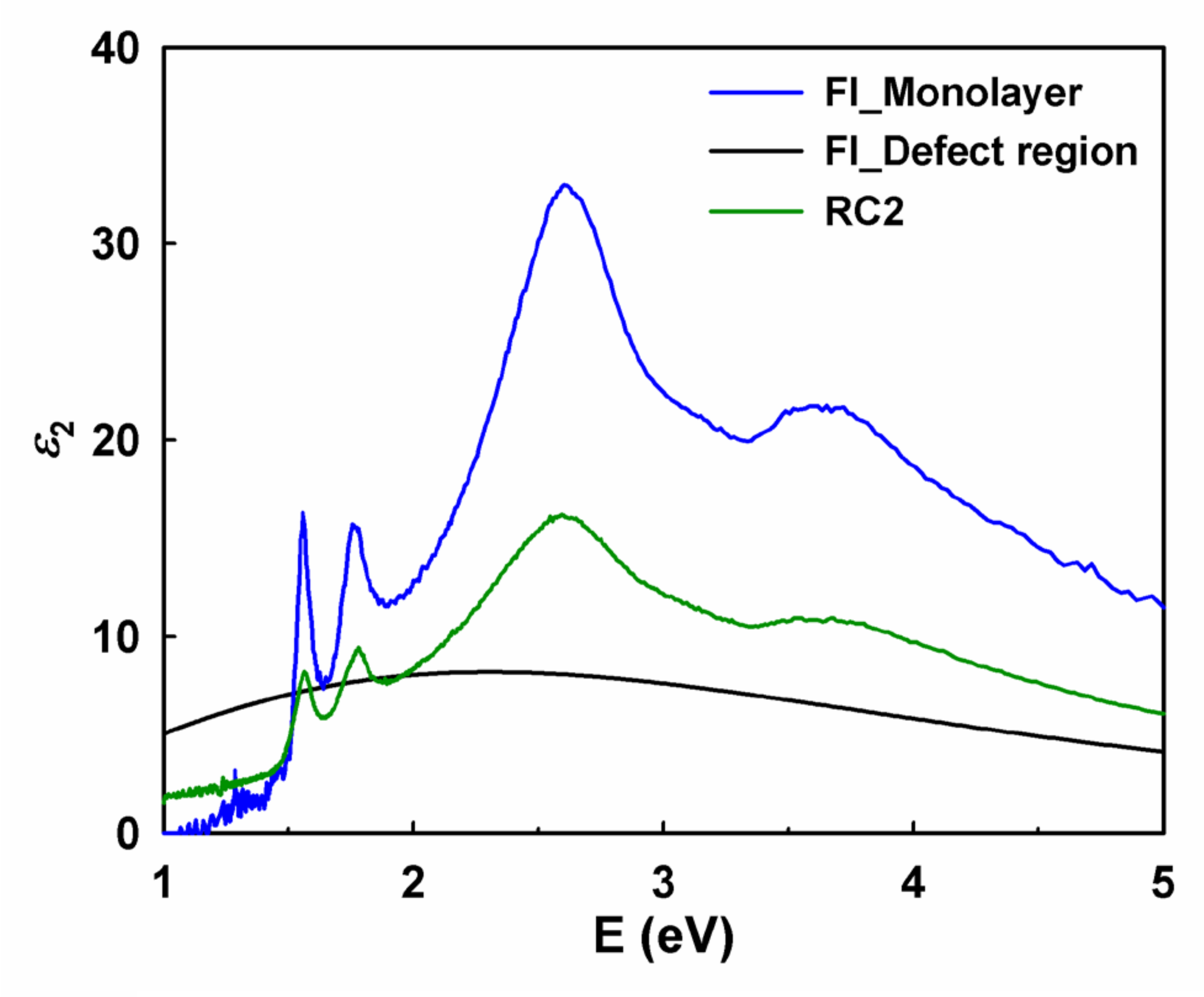

Figure S1. Imaginary parts of the dielectric spectra of monolayer $MoSe_2$ domain (blue line), defect region (black line), and submonolayer $MoSe_2$ (green line).

Figure S1 shows three dielectric spectra. The blue line is extracted from μm-scale monolayer $MoSe_2$ region measured by M2000-FI of focused beam along with analysis by point-by-point fitting approach. The black line is averaged spectrum from several incomplete $MoSe_2$ and MoO_3 area measured by M2000-FI with analysis using TL oscillator model. The green line was obtained from large mm-scale submonolayer $MoSe_2$ measured by RC2 with analysis by point-by-point approach.

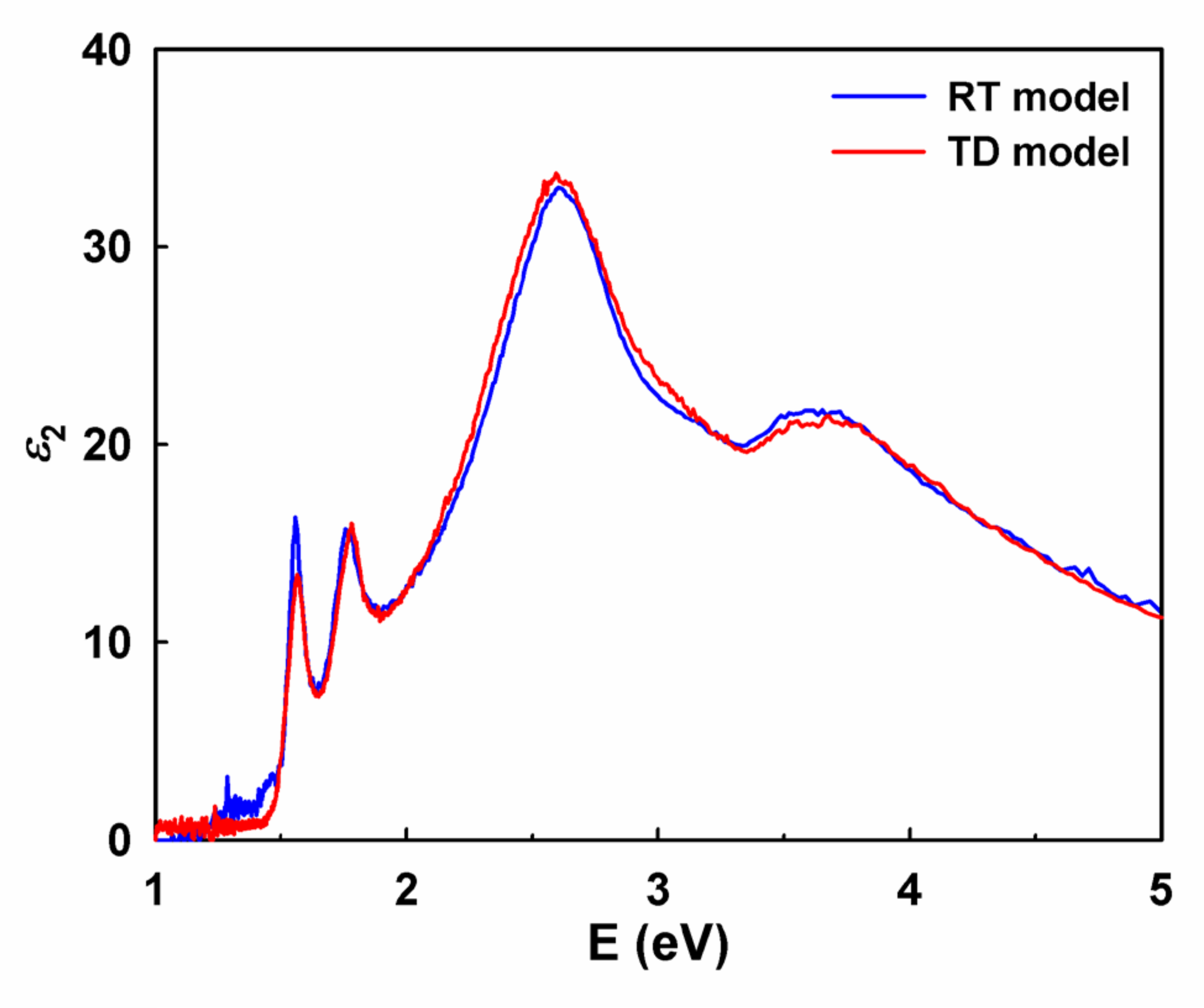

Figure S2. The comparison of Imaginary parts of ε of monolayer $MoSe_2$ at 300 K determined by RT (blue line) and TD (red line) models.

Figure S2 shows the extracted dielectric spectra obtained by two optical models. The results coincides well, confirming the validity of this analysis work. Small discrepancies are carefully examined to check whether they have any effect on the precise determination of CP energies as shown in Fig. S3 and Table S1.

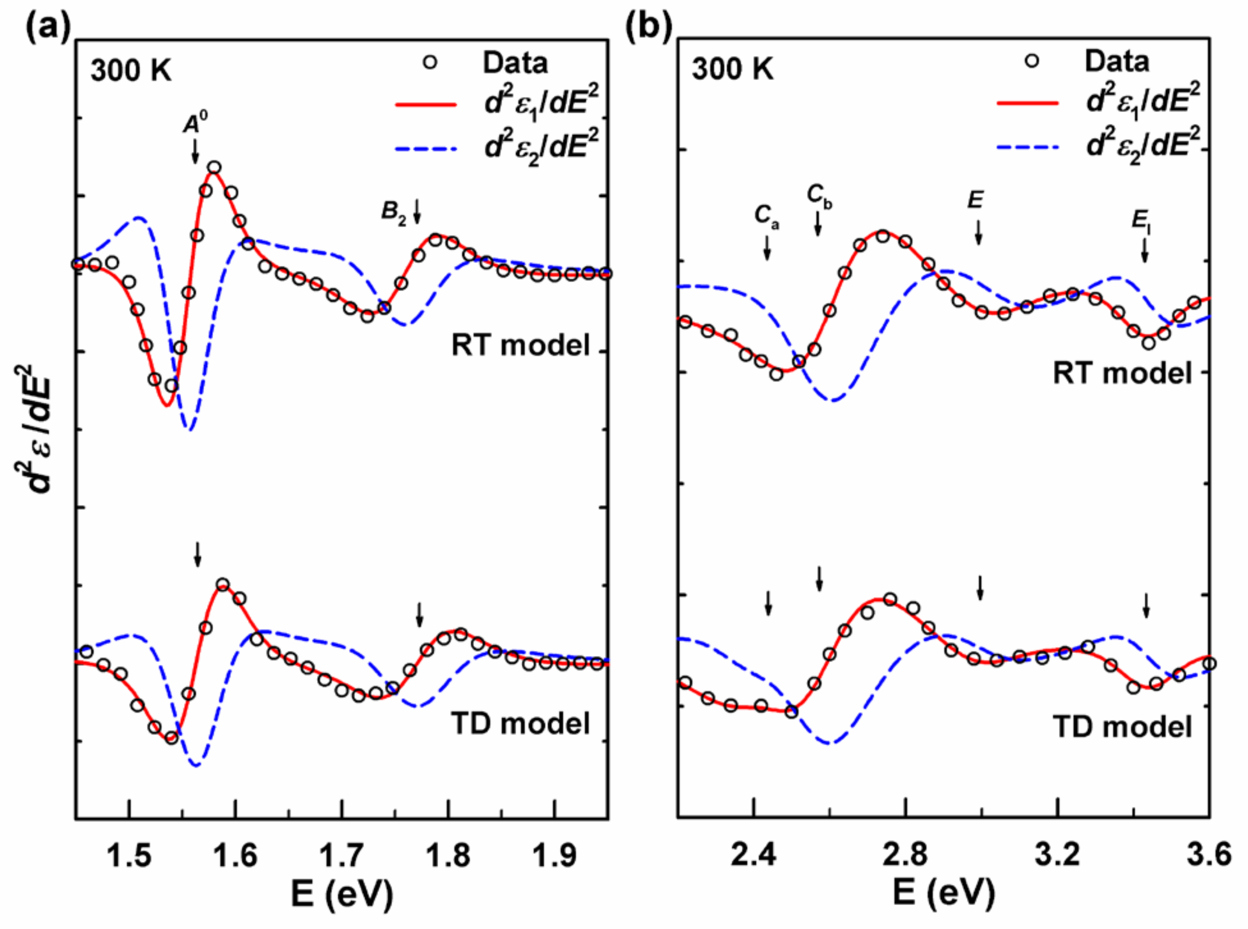

Figure S3. The comparison of the second derivatives of ε of monolayer $MoSe_2$ at 300 K extracted from RT and TD models for (a) A^0 & B_2 CPs and (b) C_a, C_b, E, & E_I CPs.

In Fig. S3, the best fits to $\frac{d^2\varepsilon_1}{dE^2}$ (red solid lines) and $\frac{d^2\varepsilon_2}{dE^2}$ (blue dashed lines) are shown. Data for only $\frac{d^2\varepsilon_1}{dE^2}$ (black open circles) is shown and number of data points are also appropriately reduced for clarity. Slight difference in Fig. S2 did not cause any notable change in the derivative spectra.

CP energies (eV)	RT model	TD model	Difference
A^0	1.55	1.56	0.01
B_2	1.76	1.77	0.01
C_a	2.42	2.43	0.01
C_b	2.59	2.57	0.02
E	3.03	3.00	0.03
E_I	3.44	3.44	0.00

Table S1. The comparison of CP energies of monolayer $MoSe_2$ at 300 K determined by RT and TD models.

Table S1 presents fit results of the CP energies obtained from two models in Fig. S3. The CP-energy differences between two models are less than 0.03 eV, which indicates that the models resulted in the identical outcome.

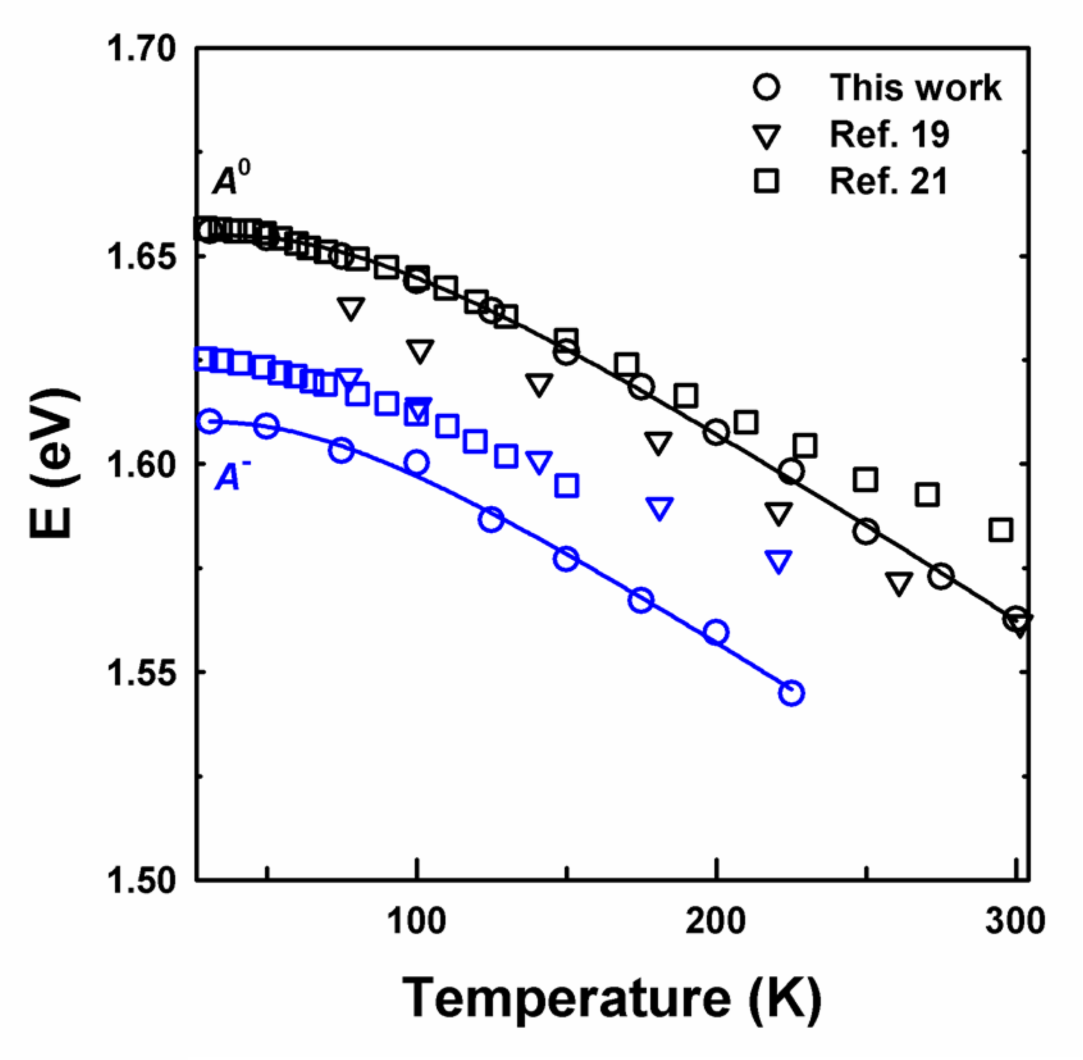

Figure S4. Temperature dependences of A^- (blue color) and A^0 (black color) CP energies (open circles) of monolayer $MoSe_2$ and the best fits (solid lines) with previously reported data in Refs. 19 (open inverted triangles) and 21 (open squares).

Figure S4 shows the comparison of temperature dependence of A^- and A^0 CP energies with reported values. A^0 CP energies coincide with the reported values of Ref. 21 from 31 to 150 K but after 150 K there are slight differences less than 0.02 eV. A^- CP energies of this work shows also 0.02 eV mismatch with the values in Refs. 19 and 21, which could be understood by the differences of experimental tools and environments as the mismatch in A^0 CP energies between Refs 19 and 21.
